# A Case of Malignant Solitary Fibrous Tumor of the Hepatic Falciform Ligament

**DOI:** 10.7759/cureus.117420

**Published:** 2026-10-04

**Authors:** Kenichiro Shimazaki, Tasuku Nagumo, Miki Kushima

**Affiliations:** 1 Department of Radiology, Showa Medical University, Tokyo, JPN; 2 Department of Pathology, Showa Medical University, Tokyo, JPN

**Keywords:** cd34 negativity, demicco risk model, hepatic falciform ligament, nab2-stat6 fusion, solitary fibrous tumor, stat6 immunohistochemistry

## Abstract

Solitary fibrous tumors (SFTs) are rare mesenchymal tumors that most commonly arise in the thoracic region. Most SFTs are benign; however, some show malignant behavior, such as local recurrence and distant metastasis. Here, we report the case of a 61-year-old man in whom an abdominal mass and multiple pulmonary nodules were incidentally detected on computed tomography (CT). Abdominal magnetic resonance imaging (MRI) showed a well-circumscribed solid tumor arising from the anterior surface of the liver that displaced the round ligament posteriorly. A gastrointestinal stromal tumor (GIST) was initially suspected; however, at laparotomy the tumor was found to arise from the falciform ligament, and a diagnosis of SFT was confirmed histopathologically. Immunohistochemically, the tumor was diffusely positive for STAT6, bcl-2, CD99, and vimentin, partially positive for cytokeratin AE1/AE3, and negative for CD34. Postoperatively, there was no local recurrence, but follow-up CT showed progressive enlargement of the bilateral pulmonary nodules and lymph nodes, and biopsy of a mediastinal lymph node confirmed metastatic SFT. The patient was transferred to a specialized cancer center for systemic therapy 13 months after surgery. To our knowledge, only two cases of SFT of the hepatic falciform ligament have been reported, and neither showed metastasis; the present case is the first with documented distant metastasis. Loss of CD34 expression and aberrant cytokeratin positivity, which have been associated with dedifferentiation and aggressive behavior in SFT, may identify extrapleural SFTs that warrant long-term surveillance for metastasis, although this observation from a single case requires confirmation.

## Introduction

Solitary fibrous tumor (SFT) is a mesenchymal tumor of fibroblastic origin that can arise anywhere in the body, including the thorax and abdomen [1]. Klemperer and Rabin first described SFT as a tumor of the pleura [2]. In the 2020 World Health Organization classification of soft tissue and bone tumors, SFT is regarded as a tumor of intermediate (rarely metastasizing) malignant potential: most SFTs are slow-growing, painless, and clinically benign, but local recurrence and/or metastasis can occur, sometimes many years after resection, and benign and malignant SFTs cannot be reliably distinguished on histological grounds alone [1,3]. Surgical resection is the mainstay of treatment, whereas chemotherapy and antiangiogenic agents are used for advanced disease [3].

Almost all SFTs harbor a *NAB2-STAT6 *gene fusion, which results from the fusion of the adjacent *NAB2* and *STAT6* genes on chromosome 12q13. In the fusion protein, the transcriptional repressor domain of NAB2 is replaced by the transactivation domain of STAT6, so that NAB2, which normally represses early growth response 1 (EGR1)-mediated transcription, becomes a constitutive activator of EGR1 target genes and drives the overexpression of growth factors such as IGF2, FGF2, and PDGFD [3]. Because the fusion protein accumulates in the nucleus, nuclear STAT6 immunostaining is a highly sensitive and specific diagnostic marker of SFT that has largely superseded the less specific markers CD34, CD99, and bcl-2 [4]. Conversely, loss of CD34 expression and aberrant cytokeratin expression have been reported in malignant and dedifferentiated SFTs [5-8].

Clinically and radiologically, features that favor malignant behavior include a large tumor size (>10 cm), heterogeneity with necrosis, hemorrhage, or cystic degeneration, infiltration of adjacent structures, avid fluorine-18 fluorodeoxyglucose (^18^F-FDG) uptake on positron emission tomography (PET), and the presence of metastases [1,5]. Histologically, hypercellularity, nuclear atypia, necrosis, and a mitotic count of four or more per 10 high-power fields (HPF) are the established criteria of malignancy, and risk stratification models that integrate patient age, tumor size, mitotic count, and necrosis are used to estimate the risk of metastasis after resection [1,5,9].

The falciform ligament is a sagittally oriented double fold of peritoneum that connects the anterior surface of the liver to the diaphragm and to the anterior abdominal wall above the umbilicus; its free inferior edge contains the round ligament of the liver (ligamentum teres hepatis) and the paraumbilical veins [10]. Primary tumors of the falciform ligament are exceedingly rare [11]. To the best of our knowledge, only two cases of SFT arising from the falciform ligament have been reported: a 50-year-old man with a 16-cm SFT containing multiple foci of histologically malignant transformation who remained disease-free five years after excision [11], and a 41-year-old man with a 6-cm fat-forming (lipomatous) variant [12]. Neither patient developed metastasis. Herein, we report a case of SFT of the falciform ligament with distant metastasis in which the tumor was negative for CD34 and partially positive for cytokeratin. The objectives of this report are (1) to describe the radiological and histopathological features that led to the diagnosis, (2) to characterize the immunophenotypic deviation from conventional SFT, and (3) to discuss the clinical course and its implications for the surveillance of extrapleural SFTs.

## Case presentation

A 61-year-old man with a history of bronchial asthma, hypertension, and obesity (body mass index, 30.5 kg/m^2^) presented to a local hospital with leg edema, which was initially attributed to a side effect of the antihypertensive agent amlodipine. Non-contrast computed tomography (CT) of the chest, abdomen, and pelvis was performed and incidentally revealed a solid mass in the upper abdomen and multiple small nodules in both lungs. The clinical course of the patient is summarized in Table 1.

**Table 1:** Clinical timeline of the present case Month 0 denotes the day of surgery; the initial CT was obtained two months before surgery. Abbreviations: ^18^F-FDG PET/CT, fluorine-18 fluorodeoxyglucose positron emission tomography/computed tomography; CT, computed tomography; GIST, gastrointestinal stromal tumor; MRI, magnetic resonance imaging; SFT, solitary fibrous tumor

Time point	Events and findings
Initial presentation (two months before surgery)	Leg edema; non-contrast chest-abdomen-pelvis CT incidentally revealed an upper abdominal mass and multiple small bilateral pulmonary nodules
Preoperative work-up	Abdominal MRI: well-circumscribed mass arising from the anterior surface of the liver and displacing the round ligament posteriorly; predominantly T1- and T2-hypointense; no restricted diffusion. Soluble interleukin-2 receptor mildly elevated (977 U/mL). GIST suspected
Surgery (Month 0)	Laparotomy: tumor arising from the falciform ligament, partially adherent to the rectus sheath; resection. Histopathological diagnosis: SFT (STAT6-positive, CD34-negative, partially cytokeratin-positive); intermediate risk (Demicco model)
Month 6 (eight months after the initial CT)	Postoperative follow-up CT: no local recurrence; enlargement of the pulmonary nodules with new nodules; enlargement of supraclavicular, hilar, hepatoduodenal ligament, and para-aortic lymph nodes
Month 7	^18^F-FDG PET/CT: multiple pulmonary nodules; FDG-avid mediastinal and supraclavicular lymph nodes
After PET/CT	Biopsy of the mediastinal lymph node: metastatic SFT
Month 13	Transfer to a specialized cancer center for systemic therapy

Abdominal magnetic resonance imaging (MRI) showed a large, well-circumscribed, ovoid solid mass protruding from the anterior surface of the liver and lying directly beneath the anterior abdominal wall. On sagittal T2-weighted images, the mass displaced the round ligament of the liver posteriorly, indicating an origin from the falciform ligament (Figure 1). Axial T2-weighted images showed the mass lying directly beneath the rectus abdominis muscles and in contact with small bowel loops (Figure 2). The mass was predominantly hypointense on both T1- and T2-weighted images, with a central area of even lower signal intensity that was initially interpreted as necrosis or edema; in retrospect, this pattern corresponded to the hyalinized collagenous stroma with edematous change found on histological examination, because collagen-rich fibrous tissue is hypointense on both T1- and T2-weighted images owing to its low proton density and short T2 relaxation time [5]. On diffusion-weighted imaging (b = 1000 s/mm^2^), the mass showed only mild hyperintensity without a decrease in the apparent diffusion coefficient (ADC), indicating the absence of restricted diffusion (Figure 2). Because of this contact with the small intestine, a gastrointestinal stromal tumor (GIST) was suspected. Laboratory findings were unremarkable except for a mild elevation of the soluble interleukin-2 receptor level (977 U/mL).

**Figure 1:**
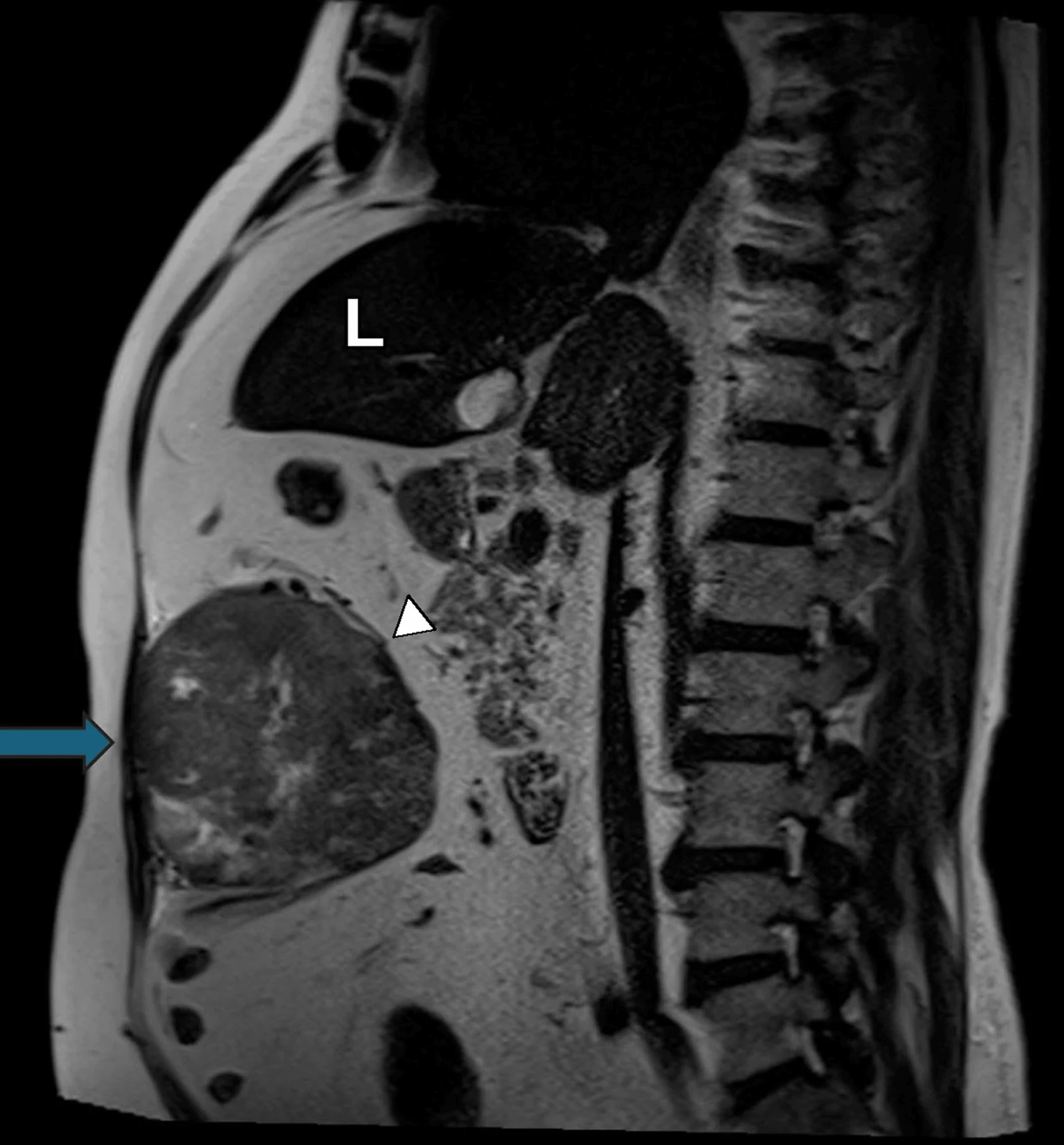
Sagittal T2-weighted MRI of the abdominal mass Sagittal T2-weighted MRI (TR/TE, 810/113 ms) shows a large, well-circumscribed, ovoid mass (arrow) lying beneath the anterior abdominal wall, inferior to the liver (L). The mass is predominantly hypointense with scattered hyperintense foci. The round ligament of the liver (arrowhead) is displaced posteriorly by the mass, indicating an origin from the falciform ligament. Abbreviations: MRI, magnetic resonance imaging; TR, repetition time; TE, echo time

**Figure 2:**
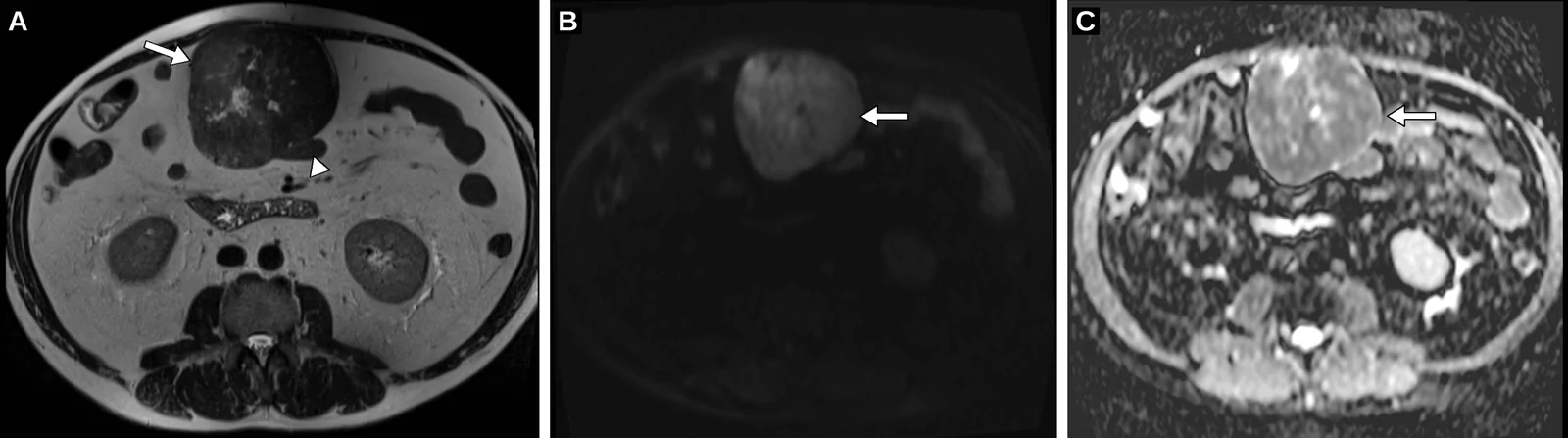
Axial MRI of the abdominal mass (A) Axial T2-weighted image shows the well-circumscribed, predominantly hypointense mass (arrow) with scattered hyperintense foci, lying directly beneath the rectus abdominis muscles in the anterior abdomen and abutting a small bowel loop (arrowhead). (B) Diffusion-weighted image (b = 1000 s/mm^2^) shows mild hyperintensity of the mass (arrow). (C) The corresponding apparent diffusion coefficient (ADC) map shows no decrease in the ADC within the mass (arrow), indicating the absence of restricted diffusion. Abbreviation: MRI, magnetic resonance imaging

Two months after the initial CT, the patient underwent laparotomy with a presumptive diagnosis of GIST arising from the small intestine. At surgery, however, the tumor was found to arise from the falciform ligament and to be partially adherent to the rectus sheath, and it was resected. The resected tumor was diagnosed histopathologically as an SFT (This is elaborated in the Pathological findings section).

Postoperative follow-up CT six months after surgery showed no local recurrence in the abdomen. However, compared with the initial CT, the bilateral pulmonary nodules had enlarged, and several new nodules had appeared (Figure 3), and lymph nodes in the supraclavicular, hilar, hepatoduodenal ligament, and para-aortic regions had also enlarged. Fluorine-18 fluorodeoxyglucose positron emission tomography/computed tomography (^18^F-FDG PET/CT) performed seven months after surgery showed multiple pulmonary nodules and FDG-avid enlarged mediastinal and supraclavicular lymph nodes (Figure 4). Biopsy of the mediastinal lymph node confirmed metastatic SFT. Thirteen months after surgery, the patient was transferred to a specialized cancer center for systemic therapy; detailed information on the subsequent treatment and outcome was not available to us.

**Figure 3:**
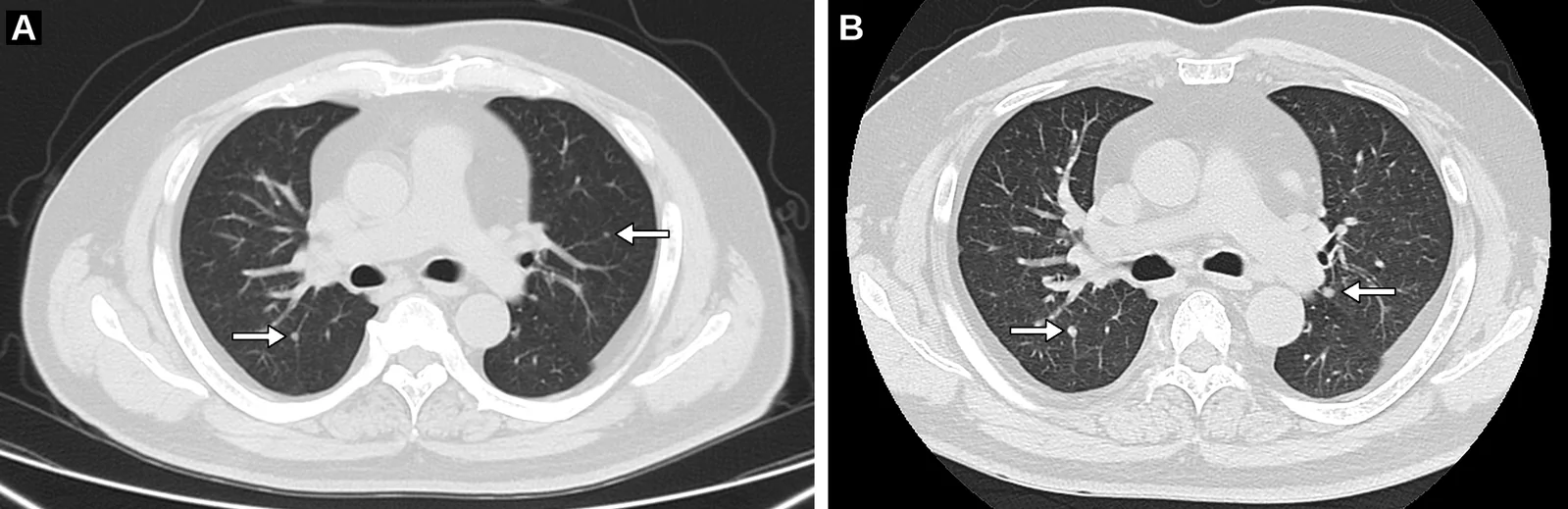
Chest CT showing the pulmonary nodules at presentation and at follow-up (A) Axial non-contrast chest CT (lung window) at the initial presentation shows small round nodules in both lungs (arrows), which were detected at the same time as the abdominal mass. (B) Follow-up CT obtained eight months later (six months after surgery) shows enlargement of the nodules and new nodules (arrows), consistent with pulmonary metastases. Abbreviation: CT, computed tomography

**Figure 4:**
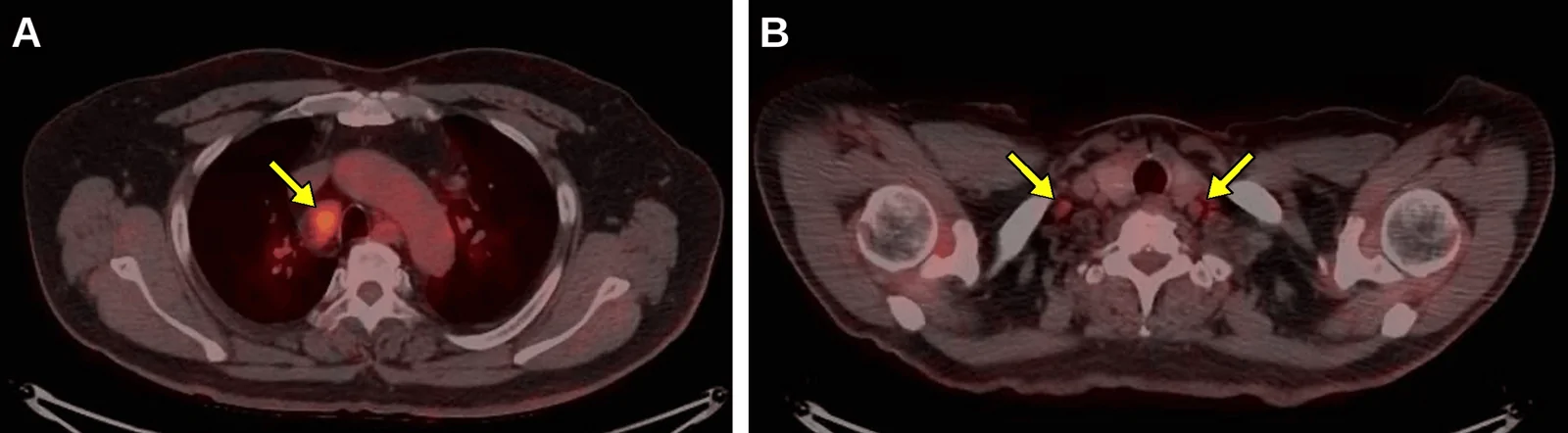
FDG PET/CT obtained seven months after surgery Fused ^18^F-FDG PET/CT images. (A) An FDG-avid enlarged mediastinal lymph node (arrow). (B) FDG-avid enlarged bilateral supraclavicular lymph nodes (arrows). The mediastinal lymph node was subsequently biopsied and confirmed to be a metastatic solitary fibrous tumor. Abbreviation: ^18^F-FDG PET/CT: fluorine-18 fluorodeoxyglucose positron emission tomography/computed tomography

Pathological findings

The resected tumor measured 12.5 × 11.0 × 8.5 cm and was grossly described as a solid, elastic-hard mass with a thin circumferential fibrous capsule. The cut surface was yellowish-white with focal hemorrhage, and no macroscopic necrosis was identified (Figure 5).

**Figure 5:**
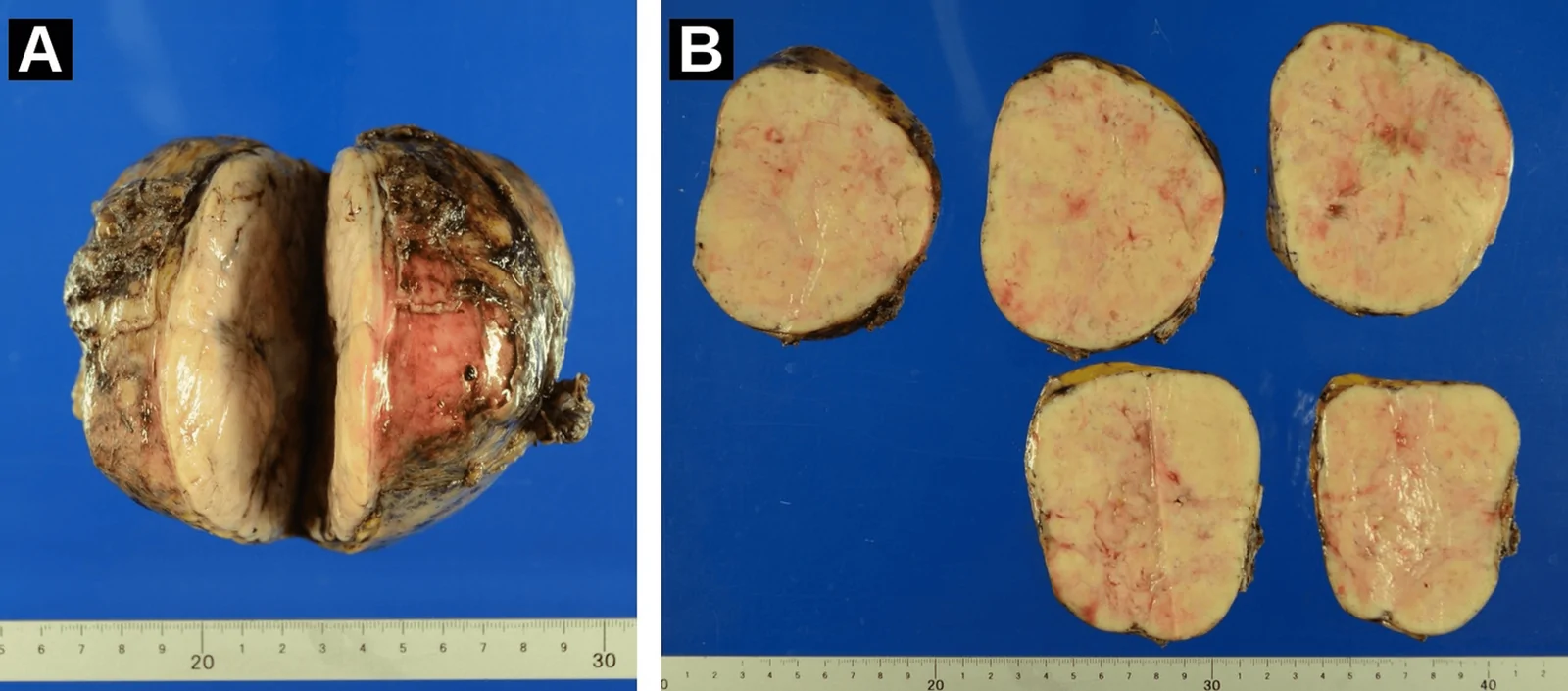
Gross appearance of the resected tumor (A) External surface of the resected tumor (12.5 × 11.0 × 8.5 cm), which is covered by a thin fibrous capsule. (B) Serial cut sections show a solid, elastic-hard, yellowish-white cut surface with focal hemorrhage and no macroscopic necrosis. Scale in centimeters.

Histologically, the tumor consisted of short spindle cells with indistinct cell borders arranged in a patternless, irregularly solid and cord-like growth pattern, with staghorn-shaped (hemangiopericytoma-like) branching vessels and a hyalinized fibrous stroma with edematous change. An epithelioid growth pattern was observed focally. The nuclei were uniform and round to oval with mild atypia; the mitotic count was three per 10 HPF, and no necrosis was identified (Figure 6).

**Figure 6:**
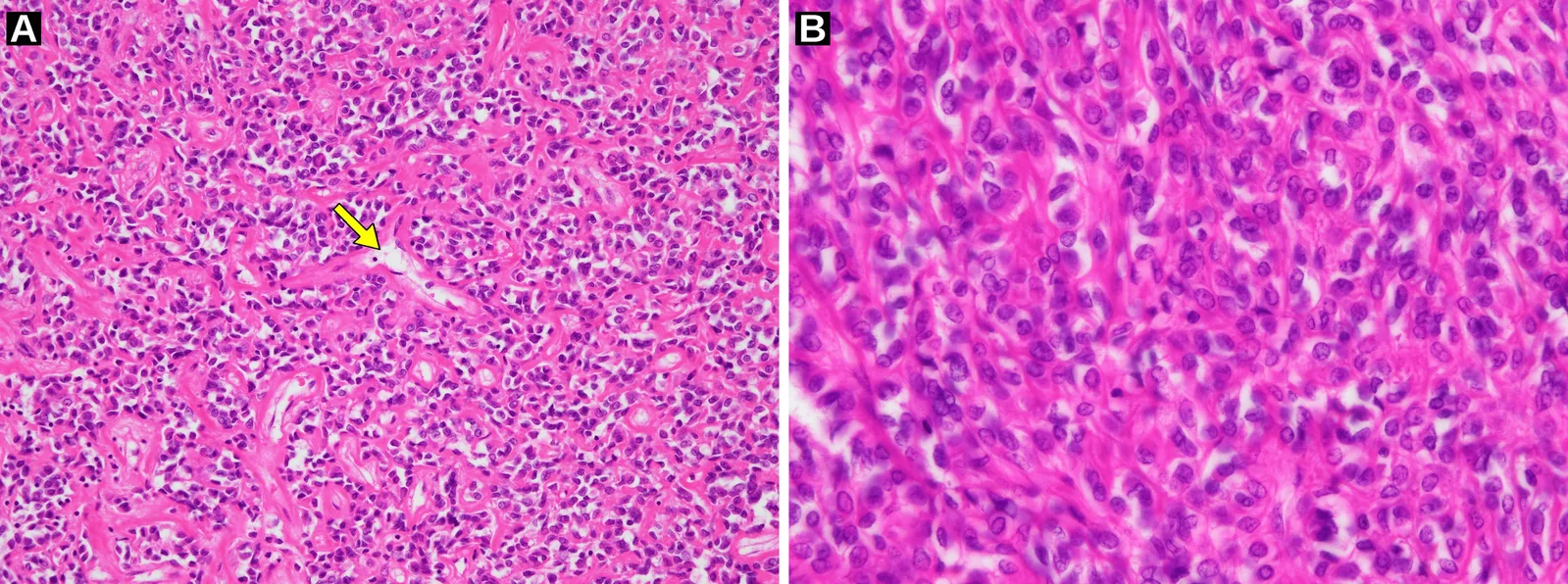
Histopathological findings of the resected tumor (A) Hematoxylin and eosin (H&E) stain, ×200. Short spindle cells with indistinct cell borders and oval nuclei proliferate in a patternless, irregularly solid and cord-like pattern. A staghorn-shaped (hemangiopericytoma-like) branching vessel (arrow) and hyalinized fibrous stroma with edematous change are seen. (B) H&E stain, ×400. The nuclei are oval and uniform with mild atypia and inconspicuous nucleoli; no mitotic figure is present in this field (overall mitotic count, 3 per 10 high-power fields).

Immunohistochemically, the tumor cells were diffusely positive for STAT6 (nuclear), CD99, bcl-2, and vimentin, and partially positive for cytokeratin AE1/AE3, epithelial membrane antigen (EMA), and CD10, whereas they were negative for CD34 (with preserved staining of the intratumoral endothelial cells as an internal control), α-smooth muscle actin, desmin, S-100 protein, c-kit (CD117), and DOG1. The Ki-67 labeling index was 6.1% (45/735 cells) (Figure 7). The immunohistochemical panel and its interpretation are summarized in Table 2. On the basis of the morphology and the strong nuclear STAT6 expression, the tumor was diagnosed as an SFT; the negativity for c-kit and DOG1 excluded GIST. According to the risk stratification model of Demicco et al. [9] (age ≥55 years, 1 point; tumor size 10 to <15 cm, 2 points; mitotic count 1-3 per 10 HPF, 1 point; necrosis <10%, 0 points), the tumor was classified as intermediate risk (total, 4 points).

**Figure 7:**
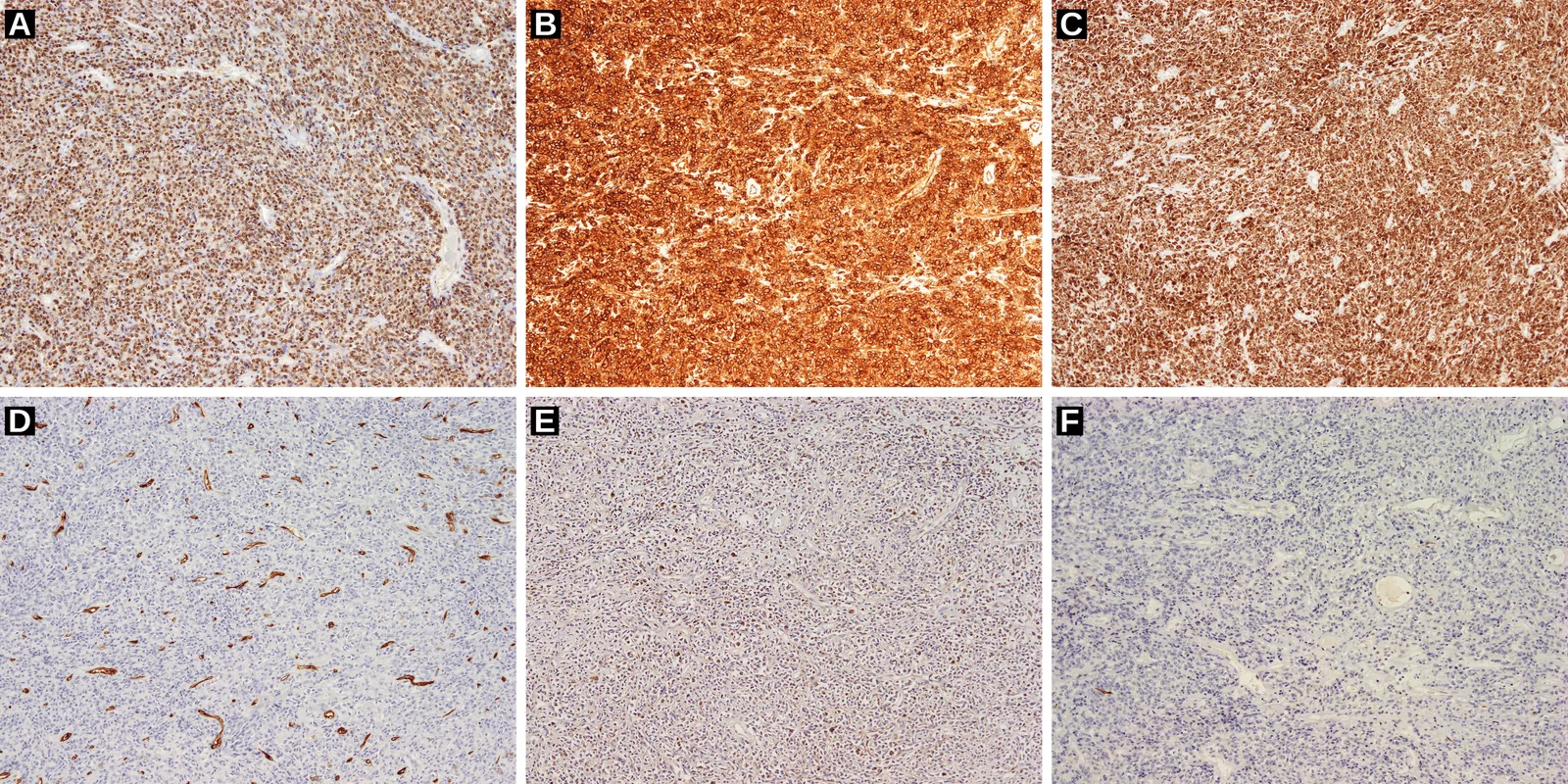
Immunohistochemical findings of the resected tumor All panels, ×100. (A) Diffuse and strong nuclear staining for STAT6. (B) Diffuse staining for CD99. (C) Diffuse staining for bcl-2. (D) The tumor cells are negative for CD34, whereas the endothelial cells of the intratumoral vessels are positive and serve as an internal positive control. (E) Partial (weak to moderate) cytoplasmic staining for cytokeratin AE1/AE3. (F) Ki-67 labeling index of 6.1%.

**Table 2:** Immunohistochemical profile of the present tumor and its interpretation Abbreviations: EMA, epithelial membrane antigen; GIST, gastrointestinal stromal tumor; SFT, solitary fibrous tumor; SMA, smooth muscle actin

Marker	Result in the present case	Interpretation and diagnostic significance
STAT6 (nuclear)	Diffusely positive	Surrogate marker of the *NAB2*-*STAT6* fusion; highly sensitive and specific for SFT; confirmatory of the diagnosis [4]
CD99	Diffusely positive	Frequently positive in SFT but nonspecific [4,13,14]
bcl-2	Diffusely positive	Frequently positive in SFT but nonspecific [4,13,14]
Vimentin	Diffusely positive	Mesenchymal marker; nonspecific
CD56	Positive	Nonspecific
CD34	Negative (intratumoral endothelium positive)	Positive in 81-95% of conventional SFTs [8]; loss of expression is associated with malignant transformation and dedifferentiation [3,5-8]
Cytokeratin AE1/AE3	Partially positive (weak to moderate)	Usually negative in SFT; aberrant expression reported in malignant and dedifferentiated SFT and a pitfall for sarcomatoid carcinoma or mesothelioma [7,8]
EMA	Partially positive	Usually negative in SFT; focal expression of epithelial markers should be interpreted together with STAT6
CD10	Partially positive	Nonspecific
α-SMA, desmin	Negative	Argue against smooth muscle and myofibroblastic tumors (e.g., leiomyosarcoma)
S-100 protein	Negative	Argues against nerve sheath tumors and melanoma
c-kit (CD117), DOG1	Negative	Exclude GIST, the main clinical differential diagnosis
SOX2	Negative	No diagnostic implication in this case
Ki-67	6.1% (45/735 cells)	Proliferation index; higher values are seen in malignant SFT [5,13]

## Discussion

The annual incidence of extrameningeal SFT is estimated to be 6.1 cases per 10 million persons, with no clear sex or racial predilection [15]. Although SFT was first described in the pleura, it can arise at any site; in a series of 110 cases, the abdomen and pelvis were the most common sites of origin (33 cases) [1]. SFTs arising from the mesentery or peritoneum are uncommon [16], and only two cases arising from the falciform ligament had been reported before the present case [11,12]. Primary tumors of the falciform ligament in general are rare and include leiomyosarcoma, lipoma, yolk sac tumor, malignant mesothelioma, and SFT [11]. In the following sections, we discuss the diagnostic considerations, the immunophenotypic deviation of the present tumor, its metastatic behavior, and the prognostic implications.

Diagnostic considerations and radiologic-pathologic correlation

The symptoms of SFT depend on its location. Abdominal SFTs are usually painless but may cause compressive symptoms such as bloating, constipation, and urinary retention, whereas SFTs of the head and neck may cause nasal obstruction, bleeding, and voice changes [17,18]. Both previously reported SFTs of the falciform ligament presented with upper abdominal pain [11,12], whereas our patient was asymptomatic and the tumor was discovered incidentally.

The imaging findings of SFT are variable and nonspecific; however, a well-circumscribed ovoid or round mass with hypointensity on T2-weighted images and avid enhancement on CT or MRI suggests the diagnosis [5]. The T2-hypointense areas correspond to collagenous stroma and fibrosis, whereas T2-hyperintense areas correspond to hypercellular, myxoid, hemorrhagic, or cystic components [5]. In the present case, the predominantly T1- and T2-hypointense mass without restricted diffusion corresponded to the hyalinized fibrous stroma seen histologically, and the sagittal T2-weighted image, which demonstrated posterior displacement of the round ligament, was the key to localizing the tumor to the falciform ligament. Tumor heterogeneity, necrosis, hemorrhage, and FDG avidity are more common in malignant SFTs [5]; in our patient, the FDG-avid lymph nodes on PET/CT were consistent with malignant behavior, and biopsy of the mediastinal lymph node confirmed metastasis.

Because the mass abutted the small intestine, GIST, the most common mesenchymal tumor of the gastrointestinal tract, was initially suspected. SFT and GIST cannot be reliably distinguished by imaging or by morphology alone, because both may present as a well-circumscribed, hypervascular spindle cell tumor in contact with the bowel; the distinction therefore rests on immunohistochemistry. In the series of Yoshida et al., nuclear STAT6 staining was present in 100% of SFTs but in only 2.5% of histological mimics and in none of nine GISTs [4], whereas GIST is characterized by c-kit and DOG1 positivity. In our case, the strong nuclear STAT6 positivity together with the negativity for c-kit and DOG1 established the diagnosis of SFT. The differential diagnosis of a cytokeratin-positive, CD34-negative spindle cell tumor of the peritoneal cavity also includes sarcomatoid mesothelioma and sarcomatoid carcinoma, in which nuclear STAT6 is negative [4,8].

Immunophenotypic deviation: loss of CD34 and aberrant cytokeratin expression

Immunohistochemistry with STAT6, CD99, CD34, bcl-2, and vimentin is essential for the diagnosis of SFT [4,13,14]; among these markers, STAT6 is the most useful because of its high sensitivity and specificity, and positive STAT6 staining was critical for the diagnosis in the present case [4]. The *NAB2*-*STAT6* fusion converts NAB2 into a transcriptional activator of EGR1 target genes, resulting in the overexpression of growth factors such as IGF2, FGF2, PDGFD, and FGFR1, which is thought to initiate tumorigenesis [3].

CD34 is expressed in 81%-95% of conventional SFTs [8], but its expression may be attenuated or lost in malignant and dedifferentiated SFTs [3,6-8]. Yokoi et al. reported that all three malignant SFTs in their series were negative for CD34 and proposed that malignant transformation is closely associated with dedifferentiation, as indicated by the loss of CD34 expression, together with p53 overexpression [6]. In dedifferentiated SFT, additional genetic events, most consistently *TP53* mutation with loss of 17p and loss of *RB1*, accompany the progressive loss of CD34, CD99, and even STAT6 expression [3]. Cytokeratin expression is unusual in SFT, but multifocal cytokeratin positivity has been described in morphologically malignant pleural and abdominal SFTs and in dedifferentiated SFT, in which it represents a diagnostic pitfall for sarcomatoid carcinoma or mesothelioma [7,8]. In the present case, cytokeratin was expressed only partially, and an epithelioid growth pattern was observed focally; aberrant epithelial marker expression in SFT may therefore be related to epithelioid or dedifferentiated morphology, as has been observed in other soft tissue tumors. Loss of CD34 expression and cytokeratin positivity have also been reported to correlate with high-grade histology and an unfavorable outcome in pleural SFTs [5]. Our tumor showed both features and had metastasized; however, we emphasize that this association is an observation from a single case rather than a validated prognostic marker, particularly because the primary tumor showed only mild atypia, a mitotic count of 3 per 10 HPF, and no necrosis, and because metastasis was already present at diagnosis.

Metastatic behavior

Approximately 80% of SFTs are cured by surgical resection [3], but 5-10% of cases show local recurrence and/or metastasis even after surgery [7], and in a referral series of 110 extrameningeal SFTs, 26% of the patients with follow-up developed metastasis, with 5- and 10-year metastasis-free rates of 74% and 55%, respectively [1]. In that series, the lung/pleura was the most common site of metastasis, followed by the liver, bone, peritoneum, brain, and soft tissue, and the median time to the first metastasis was 36 months (range, four to 228 months) [1]. The mortality of extrapleural SFT is relatively low overall [4,6,13], but patients who present with unresectable metastatic disease have a poor prognosis, with a median survival of 19 months [1]. Dedifferentiated SFT is the fastest-growing subtype and carries the highest risk of distant metastasis [3]. Once advanced or metastatic disease develops, systemic therapy has limited efficacy: the median progression-free survival is 3.5 months with anthracycline-based chemotherapy [19] and 9.7-10.8 months with temozolomide plus bevacizumab according to the Choi and Response Evaluation Criteria in Solid Tumors (RECIST) criteria, respectively [20], and antiangiogenic agents are now favored over conventional chemotherapy [3]. Postoperative radiotherapy reduces local recurrence but does not improve overall survival [3].

In our patient, the pulmonary nodules were already present on the initial CT and, together with the mediastinal, supraclavicular, hilar, and abdominal lymph nodes, enlarged progressively over the following year, consistent with hematogenous pulmonary and lymphatic spread from the primary tumor; the mediastinal lymph node metastasis was confirmed histologically. Lymph node metastasis is uncommon in SFT, and the combination of nodal and pulmonary spread at presentation underscores the aggressive biology of this tumor. The lungs and pleura, the metastatic sites in our patient, are also the most frequently reported sites of metastasis in SFT [1].

Comparison with previously reported cases and risk assessment

Table 3 compares the present case with the two previously reported SFTs of the falciform ligament [11,12].

**Table 3:** Comparison of the present case with the previously reported solitary fibrous tumors of the falciform ligament *Partial (weak to moderate) positivity. **Risk group according to the risk stratification model of Demicco et al. [9], which is based on patient age, tumor size, mitotic count, and necrosis; for the case of Dickfos et al. [12], necrosis was not reported, but the total score is ≤3 (low risk) irrespective of necrosis. Abbreviations: CK, cytokeratin; EMA, epithelial membrane antigen; HPF, high-power fields; IHC, immunohistochemistry; M, male; SMA, smooth muscle actin

Reference	Age (years)/sex	Presentation	Treatment	Tumor size (cm)	IHC positive	IHC negative	Necrosis	Mitotic count (/10 HPF)	Risk group**	Recurrence/metastasis	Outcome
Gidwani et al. (2004) [11]	50/M	Acute severe upper abdominal pain; epigastric mass noted 8 years earlier	Surgical excision	16 × 12 × 10	CD34, CD99	Not reported	Present (focal, in cellular nodules)	5	High	None	Disease-free at five years without adjuvant therapy
Dickfos et al. (2016) [12]	41/M	Cramping epigastric pain and reflux; palpable epigastric mass	Surgical excision	6.1 × 4.8 × 3.1	STAT6, bcl-2, CD99, S-100, CD34 (patchy)	SMA, desmin, CD117, DOG1 (*MDM2* amplification and *DDIT3* rearrangement absent)	Not reported	≤2	Low	None	Annual ultrasound surveillance planned (fat-forming variant)
Present case (2025)	61/M	Asymptomatic (incidental finding)	Surgical resection	12.5 × 11.0 × 8.5	STAT6, CD99, CD56, vimentin, bcl-2, CK AE1/AE3*, EMA*, CD10*	CD34, α-SMA, desmin, CD117, DOG1, SOX2, S-100	Absent	3	Intermediate	Lung and lymph node metastases (present at diagnosis; histologically confirmed)	Progressive metastatic disease; transferred for systemic therapy at 13 months

Histologically, the present case was similar to the two previously reported cases in showing diffuse positivity for CD99 and STAT6 (STAT6 was not examined in the case of Gidwani et al.), but it differed in being negative for CD34, which was positive in both previous cases [11,12]. Clinically, both previous patients presented with upper abdominal pain, whereas our patient was asymptomatic. Neither of the previous patients developed metastasis: the patient of Gidwani et al., whose tumor contained multiple foci of histologically malignant transformation with focal necrosis and five mitoses per 10 HPF (high risk according to the model of Demicco et al.), remained free of disease five years after excision without adjuvant therapy [11], and the patient of Dickfos et al., whose tumor was a fat-forming variant with a low mitotic count, was scheduled for annual ultrasound surveillance [12]. In contrast, our patient had pulmonary and lymph node metastases at presentation despite less alarming histological parameters (mild atypia, three mitoses per 10 HPF, and no necrosis) and an intermediate-risk score. This discordance illustrates that the metastatic potential of SFT is not always predictable from the histology of the primary tumor and supports the view that loss of CD34 and aberrant cytokeratin expression may identify a subset of SFTs with more aggressive behavior.

Prognostic implications and surveillance

Risk stratification models based on patient age, tumor size, mitotic count, and necrosis [1,9] are used to estimate the risk of metastasis after resection, and the present tumor fell into the intermediate-risk group. These models, however, do not incorporate the immunophenotype, and our case, together with previous reports [5-8], suggests that loss of CD34 and aberrant cytokeratin expression may flag tumors with a higher metastatic potential than the histological score alone would predict. Because metastasis of SFT can occur many years after resection [1], long-term surveillance is recommended for all resected SFTs. For extrapleural SFTs with an atypical immunophenotype, such as CD34 loss and cytokeratin positivity, closer follow-up including chest CT (the lung being the most frequent site of metastasis) at shorter intervals may be prudent, although the optimal interval remains to be defined. Integration of imaging, histopathology, and immunophenotyping into an individualized risk assessment may help to tailor the surveillance strategy for each patient. The limitations of this report are its single-case design and the lack of detailed information on the systemic treatment and outcome after the patient was transferred.

## Conclusions

We report an extremely rare case of malignant SFT arising from the hepatic falciform ligament, the first with documented distant (pulmonary and lymph node) metastasis, in which the tumor was negative for CD34 and partially positive for cytokeratin. Although a single case cannot establish these immunophenotypic features as prognostic markers, this case suggests that extrapleural SFTs showing CD34 loss or aberrant cytokeratin expression may behave aggressively even when the histological risk score is not high, and that such patients warrant long-term surveillance for metastasis. This case also highlights the importance of integrating radiology (in particular, the relationship of the mass to the round ligament), histopathology, and immunophenotyping (STAT6, CD34, and cytokeratin) in the accurate diagnosis and risk assessment of rare soft tissue tumors of the peritoneal ligaments.
